# Practice of Medicine

**Published:** 1859-01

**Authors:** 


### PRACTICE OF MEDICINE.

1. PRACTICAL OBSERVATIONS ON CARDIALGIA. By *Professor Fenger.*—Professor Fenger states that this disease is of very frequent occurrence in Copenhagen, especially among the maid-servants. It is also a frequent symptom in other diseases, and then being of varying severity and combined with numerous other symptoms, may not take the leading rank. Among such diseased conditions are various fevers, inflammatory affections of the chest or abdomen, cholera, and other diseases, attended with great discharges, various nervous affections, as hysteria, epilepsy, tetanus, and some forms of mental disease. In these cases the cardialgia disappears as the original disease diminishes, and at most calls for palliatives. Not infrequently, also, where the cardialgia appears as a leading symptom, it is found to be masking other diseases, the true seat of which may be in other parts of the body. Thus, it is a frequent symptom in chlorosis, and acquires sometimes such severity as to be the chief one concerning which the patient complains. The pain in such cases is not usually continuous, and is relievable by palliatives; but sometimes it is very obstinate, returning again and again, until a long course of iron has been administered. Of all the preparations of this, the *ferrum desoxydatum,* two to four grains three times daily, in the form of pastille or chocolate, is the best. In *acute poisoning* by caustics, metallic substances, or narcotics, cardialgia is a very common symptom, and in *chronic poisoning* by arsenic, lead, etc., it may constitute a leading one. It is also very common in chronic alcohol poison. In all such cases a rigid diet, long maintained, and the absolute prohibition of alcohol, does the greatest service, especially if the patient can keep his bed the while. Cardialgia is observed in many of the dyscrases, especially *gout*, constituting with nausea and vomiting a dangerous anomalous form of the disease, which has been regarded as a metastasis of gout to the stomach. Chronic disease of some organs at a distance from the stomach may give rise to cardialgia, the proper symptoms of these being so slight as to render the diagnosis difficult; and disorders of the functions of the *uterus* are especially liable to induce it. Whenever the cardialgia is combined with much leucorrheal discharge we must always well examine the condition of this organ. When the cardialgia seems connected with anæmia and menstrual disturbance, the *tinct. ferri muriat.* is very useful. It is sometimes a symptom of *hysteria;* but it is an error to consider it often as a hysterical disease.

In the male subject cardialgia is sometimes a symptom of *onanism*, and then it is distinguished by its severity and obstinacy. When the symptoms of onanism are not quite decided, a bougie should be passed, a practice which few onanists can bear, owing to the suffering it causes. When great pain and irritability is thus shown, and no stricture or gonorrhœa is present, it is an excellent sign of onanism having been practiced for some time. Dr. Fenger, in these cases, applies the nitrate of silver to the urethra. Cardialgia is also met with in chronic diseases of the *kidney;* and in all obstinate cases, accompanied by wasting and want of appetite, the condition of the kidney should be inquired into. It is a frequent masking symptom in affections of the *chest*, and especially in the chronic form of phthisis in the aged. In diseases of the *brain* and *spinal marrow*, there are usually characteristic symptoms besides the cardialgia; but sometimes, in disease of the spine, the pain at the epigastrium or side is so great as to draw off attention from the spine itself.

Although, as seen above, the *digestive organs* are not necessarily the seat of disease in cardialgia,—in most cases they are so. Blows on the epigastrium, as also any diseased conditions that lead to altered position or forced traction of the stomach, may give rise to obstinate cardialgia. It may also result from compression by adjacent organs, as in various diseases of the viscera, pregnancy, or distention of the intestinal canal by air or excrement. This last is a more frequent cause of the affection than is suspected. Organic diseases of the stomach are usually attended with cardialgia.

Finally, there are cases of cardialgia, in which the most careful examination fails to detect any other disease, *idiopathic cardialgia.* As Huss has already shown, this is one of the most common diseases among the common people of Sweden, and indeed throughout the North of Europe. In Copenhagen, it is especially met with among maid-servants; and the want of sufficient nutriment, and the too great use of brandy and coffee, to which Huss assigned the prevalence of the disease, cannot be attributed to them. One of the principal accompaniments is the excessive *epigastric sensibility*, which in some cases even continues during the absence of the pain. The author has often found the affection following, in maid-servants, great bodily exertion, especially of the muscles of the arms, as in washing, lifting heavy bodies, etc.; and he believes the pain is often seated in the nerves of the muscular parietes of the thorax. However this may be, in these cases, mere repose in bed for a week or fortnight suffices for a cure, even without medicine; or the same end may be obtained by putting one arm in a sling, and forbidding all exertion. In the severest cardialgia, in connection with chlorosis, such repose in bed is highly useful. When such rest is impossible, and the pain is severe, sinapisms or flying blisters are of use, employing leeches or cupping when spinal irritation is present. Another accompanying symptom is a *spasmodic contraction of the abdominal recti muscles*, which is sometimes constant, and at others is produced by touching the epigastrium. When this is supposed to be due to inflammatory irritation of subjacent organs, blood-letting is indicated; but when this is not the case, hot bread poultices give remarkable relief. *Epigastric pulsation* is another very common symptom in cardialgia; but Dr. Fenger has been unable to make out any determinate connection between it and the causes of the cardialgia. It occurs in both sexes and at different ages, and in the most diverse forms of the disease. It usually indicates obstinacy of the affection. When severe the patient should keep in bed; and local bleeding, the long maintenance of superficial suppuration, and the internal use of morphia are very useful. Another symptom, just as common and as enigmatical as this, is a *vaulting of the epigastrium outwardly*. As a general rule, the patients complain that they cannot bear the pressure of their clothes. Sometimes the symptom is only transitory, as after eating, during constipation, or during severe paroxysms of pain; but in other cases it is almost always present. The vaulted form, usually confined between the umbilicus and sternum, sometimes extends very much farther. Dr. Fenger does not admit that it is a tympanites, for it may remain for months uninfluenced by great discharges of wind. The aggravated form of the disease is infrequent, and chiefly occurs in combination with hysteria, in young women whose sexual functions are retarded.

Our treatment of cardialgia must be guided by our knowledge of its cause. Among the palliatives, ethereal and balsamic medicines are, according to the author, of no use; and he has no very great opinion even of bismuth given in doses of from five to ten grains, four times a day. There is little certainty either in the operation of nux vomica or strychnine, hyoscyamus or the aqua laurocerasi. In certain cases all these means are useful, but it is difficult to determine which are the cases. Where the pain takes on at all an intermitting character quinine is useful. Where the pain comes on daily at indeterminate times, and is attended with great prostration of strength, and from its continuance, with ill effects upon the general health, the author has found the nitrate of silver of use, commencing with one-eighth of a grain three times a day. When cardialgia is connected with vomiting after meals, and is not dependent on organic disease, a rigid diet constitutes the best treatment, and many cases have been relieved by giving milk alone, the patient only taking small quantities, and keeping in bed. Where hyperæmia of the stomach is present, local bleeding or derivatives should be employed.—*Schmidt's Jahrbücher*, band 97, pp. 317–322. (*Medical Times and Gazette*, Nov. 13, 1858.)

2. ON THE TREATMENT OF CROUP. By *Dr. Luzsinsky*.—Dr. Luzsinsky, Medical Director of the Children's Hospital at Vienna, after animadverting upon the discrepancy of opinion which prevails concerning the nature and treatment of croup, observes that it is essentially an inflammatory affection of the mucous membrane of the air-passages. Still, it is not a mere local affection of these, inasmuch as croupy exudations are also often found upon other parts of the external or internal surface; so that it arises from a peculiar condition or *crasis* of the blood giving rise to pseudo-membranous deposits, and is a mere local expression of a general diathesis. The spasm, which plays so important a part in the disease, is a secondary condition excited by this inflammation.

Speaking of the symptoms, the author observes that the true croupy tone, bearing the most striking resemblance to the barking of a young dog, once heard can never be forgotten. Some practitioners state that this peculiar cough is proper to certain children in every catarrh, and Mauthner says that the cough in short-necked children easily takes on this croupy character in simple bronchial catarrh, as also in children suffering from worms. These statements Dr. Luzsinsky has never been able to verify during fifteen years' observation of many thousand children. The warning given by the child's hoarseness and croupy tone is too often overlooked by friends and practitioners, or many more children might be saved.

The following are the indications of treatment:—

1. *To act upon the peculiar crasis of the blood.*—This is indeed unconsciously done by almost all the means that have obtained renown, as they all agree in the final effects of diminishing the plasticity of the blood. This is the case with mercurials, antimonials, sulphate of copper, and liver of sulphur. The influence of these latter articles, however, as solvents and alteratives, is too feeble, while their emetic properties in the early stages of croup produce mischievous concussion of the brain and larynx. Mercurials have the disadvantage of exciting diarrhœa or salivation, and when employed to a sufficient extent to operate upon the crasis, may themselves produce a mischievous contamination of that fluid,—a mercurialismus. Alkalies, on the other hand, without being accompanied with any of the above disadvantages, possess great fluidifying power over albuminous and fibrinous products. They have been long employed in diseases depending upon a hyperplastic condition of the blood, and were recommended strongly by some of the older writers in pleuropneumonia and croup. Dr. Luzsinsky published in 1855 and 1856 an account of the excellent results that followed his employment of carbonate of potash; and since then these results have been confirmed by other French and German practitioners. Nevertheless, the old practice of leeching is still continued by the great bulk, and is followed by a mortality of from sixty to seventy per cent. Caustic potash is the form of alkali which of all others most strongly possesses the antiplastic power, but even in the highest state of dilution it proves too irritating to the digestive organs. As a carbonate it is far milder in its operation, and can be administered in large and long-continued doses. When continued too long diarrhœa and excessive fluidity of the blood are the results if it be not suspended. Soda is milder both in taste and influence; and the bicarbonates are only suitable for slight cases. The doses of the carbonate of potass or soda are from one-half to ʒii per diem, dissolved in water and sweetened with syrup. The alkali is continued until the cough becomes easier and looser, when usually a plastic, puriform mucus is expectorated.

2. *To prevent the localization of the inflammation in the larynx.*—For this purpose it has been the habit to apply leeches. How little was to be expected from this may be judged of from the extremely slight connection that exists between the blood-vessels of the skin and those of the mucous membrane of the larynx. The author regards depletion of any kind as injurious; and at the recent meeting of the German physicians at Bonn, the younger practitioners all were found to hold the same views. Cold would seem *a priori* the best means for preventing or suppressing this inflammation; and Lauda has frequently found the hydropathic application of this successful. Dr. Luzsinsky also, in former years, frequently resorted to this, which, however, requires great precautions in respect to the accompanying diseases, the individual constitution of the patient, and the prejudices of the friends. The best means of applying cold is by keeping the base of the neck, as low down as the sternum, covered with cloths wetted in cold, and afterwards in iced-water, keeping the rest of the body warm, and assiduously administering small quantities of cold milk and water. This practice is to be continued for from one to three days, when the cough becomes looser and the respiration easier. The cold is then to be gradually decreased, and more nourishment administered. Warm cataplasms are very objectionable, encouraging determination of blood to the parts affected, obstructing the breathing, or rendering the child liable to chills. Reasonable and useful as is the application of cold, invincible obstacles are sometimes offered to it, and we must then resort to derivative applications in the form of blisters, placed just above the sternum. To this means the author attaches great importance. The surface of the blister soon becomes covered with the diphtheritic membranes. When these appear and have frequently to be removed it is a good sign; while when they are absent, and the secretions from the blistered surface defective, the child is usually lost.

3. *To relieve the spasm of the larynx.*—For the relief of the spasmodic symptoms exhibited by the distressing cough, the impending suffocation, and the great restlessness, opiates are of great service. They are so especially at the commencement, by relieving the severe cough and procuring repose for the larynx.

4. *The destruction or expulsion of the pseudo-membranes.*—The destruction of the pseudo-membranes has been attempted by means of various caustics; and of all these, the author has found the nitrate of silver of the greatest service. A solution of eight to sixteen grains to the ounce should be carefully applied by means of a pencil, several times a day, as low down toward the larynx as possible. When the false membranes begin to loosen and separate in the larynx, or when this is filled with plastic mucus or puriform membrane, and the child, unable to expectorate, is threatened with suffocation, emetics, which at an early stage can only do harm, are now indicated; of these the author prefers the cupri sulph., giving from two to eight grains in two ounces of water and one ounce of syrup, in teaspoonful doses every quarter or half hour, until vomiting is produced. If the carbonate of potass has been employed at an earlier stage, the vomiting will usually cause the discharge of plastic puriform mucus, or, more rarely, of pseudo-membranes.

A child treated according to these indications, freed from its croup, requires little after-treatment beyond diet; and recovers much more rapidly than when he has been weakened by bleeding, emetics, and purgatives, and his juices have become impregnated with mercury. As to tracheotomy, the author has no personal experience, and evidently he does not think well of it.

While during three years among 15,000 cases of children's diseases Dr. Luzsinsky met with but 30 cases of croup; in another three years he met with 60 cases among 23,000 patients. This he attributes to the reputation his successful treatment of the disease had acquired for him. Of these 90 cases, 55 occurred in boys, and 35 in girls, and the ages were as follows:—Under one year, 11 cases; from one to two, 16; from two to three, 16; from three to four, 8; from four to five, 9; from five to six, 15; from six to seven, 14; and nine years old, 1. With respect to the severity of the symptoms, these cases may be divided into three groups. The *first* is characterized by a hoarse voice, by a short, rough, barking cough, difficult respiration, indistinctness of respiratory murmur, with occasional whistling, more or less fever, and constancy of symptoms. The 36 cases of this group all recovered, because the disease was energetically attacked from the commencement. It is a class of cases calling especially for attention, because it often follows measles and influenza. Great care was taken in ascertaining that these were cases of true croup; and that every case exhibiting hoarseness with a rough cough and fever was not so designated, is shown by the fact that 100 cases were left unnoticed, under the designation catarrh of the larynx. The *second* group is distinguished by a weak, thin, screeching voice, a soundless, tubular cough, labored respiration, a very feeble and hissing respiratory murmur, and great restlessness. In forty-three patients the narrowing of the larynx had reached this point. It comprehended partly cases in which the early symptoms had been disregarded, or had not yielded to the means employed, and partly those in which the most suitable means, promptly applied, failed to check the progress of the disease. Of these cases nine died and thirty-four recovered. In five of these the friends utterly neglected the means advised. The *third* group is marked by loss of voice, mere whispering remaining, a dry, suffocating, scarcely audible cough, and a high degree of orthopnœa, the respiratory murmur being inaudible, and strangulation seeming imminent. In this condition were eleven cases, of which five recovered and six died. The disease had proceeded with such rapidity as to leave no time for the operation of remedies, or the pseudomembranes had already formed when the children were brought in. In three of the recoveries suffocation seemed imminent, and the children were only saved by the active employment of the nitrate of silver.

As the general result of the ninety cases, seventy-five recovered and fifteen died. In the first stage of the disease, all the thirty-six recovered; in the second, thirty-four of forty-three; and in the third, five of eleven. The conclusion to be drawn is, that the treatment should be prompt and active, before the crasis of the blood has become completely developed, and the inflammation localized.—*Journal für Kinderkrankheiten,* band xxix. pp. 155, 176.

[In a subsequent number of the *Journal,* (b. xxx. p. 209,) Dr. Hauner, Director of the Children's Hospital at Munich, enters into a criticism upon Dr. Luzsinsky's opinions, and concludes his paper with the following aphorisms, which, in a forthcoming work, he intends developing at full length:—1. True croup

(laryngeal croup) is a disease proper to childhood, and its cause is chiefly to be sought in the organization (the period of development) of the larynx at this period of life. 2. The anatomy and physiology of the larynx sufficiently explain the nature of croup. 3. It cannot be shown that croup is connected with any peculiarity of the blood-crasis. 4. True croup always commences in the larynx, and often passes downward to the trachea, etc.; but it never passes upward. 5. Laryngeal croup is characterized by a pseudo-membrane of more or less extent. 6. Laryngeal croup is to be carefully distinguished from diphtheritic croup, the latter always depending upon a peculiar blood-crasis, as seen in other organs of enfeebled individuals. 7. Diphtheritic croup is almost always secondary, and is not essentially different from croup in and after acute exanthemata. 8. The diphtheritic form begins, as a general rule, in the fauces, uvula, tonsils, etc., and extends hence downward. It is very rare for it to commence in the larynx or trachea. 9. A laryngeal catarrh may simulate laryngeal and diphtheritic croup very closely, but in it there is no formation of pseudo-membrane. 10. Such cases are very frequently mistaken for true croup. 11. There is no specific remedy in true croup, the treatment having to be adapted to the individual cases. 12. Emetics, cold, blood-letting, mercury, etc. are the means, adapted to special cases, that must be relied upon. 13. In certain cases of true croup an operation is desirable. 14. Diphtheritic croup requires for its treatment cauterization, emetics, alkalies, and corroborants; but calomel, bleeding, blisters, or purgatives should never be employed. 15. Tracheotomy is seldom advisable, especially on account of the liability to return of the diphtheritic process. 16. When performed it must be followed by cauterization. 17. The severest laryngeal catarrh yields, as a general rule, to antiphlogistics and suitable regimen. The favorable results obtained in many such cases have been set down as examples of the cure of croup.]—*Medical Times and Gazette*, August 7, 1858.

3. On the Introduction of a Tube into the Larynx in Croup. By *M. Bouchut.*—M. Bouchut has laid before the Académie de Médecine a proposition to treat croup by introducing a silver tube deep into the larynx. Having ascer. tained the facility with which this may be done upon the dead subject, he put the practice into force in two cases of membranous croup brought into the Sainte Eugénie Hospital during the virulent epidemic that has reently prevailed in Paris. He employs three descriptions of instruments: a small curved catheter, open at both ends, as a director for the tube; a straight cylindrical tube, one and a half or two centimetres long, furnished at its upper part with two projecting rims (one placed around its orifice and the other at six millimètres below) and pierced with a hole for the passage of a retaining silk; and a protecting sheath for the index finger or dental dilator. Having ascertained on passing the tube in the subject that it entirely entered the larynx, its upper margin being placed below the superior corda vocalis, the inferior corda fitting into the space between the two rims of the canula, and consequently above the lower rim corresponding to the internal surface of the cricoid cartilage, M. Bouchut employed it in the case of a little girl brought in with diphtheria and croup in the stage of asphyxia. The canula remained *in situ* during thirty-six hours without inducing suffocation or intefering with the functions of the epiglottis, and the symptoms of asphyxia disappeared. The larynx was thus freed from the obstructing false membranes, and the croup must be considered as having been cured. The child, however, died from the diphtheria and from a lobular pneumonia set up the night after the tube was introduced. The second case occurred in a boy three and a half years old, in whom symptoms of asphyxia had commenced. Improvement soon took place, false membranes of a large diameter being discharged through the tube, which remained *in situ* during forty hours, with the exception of one temporary removal, and never becoming displaced during coughing. At the end of this period, the fits of suffocation and asphyxia, which had been kept off for two days by means of the tube, returned; and the Internes, believing death to be imminent from obstruction occurring below the glottis, performed tracheotomy. Some false membranes were thus removed, and the tube was found to have remained in its place unobstructed.

M. Bouchut considers that these two facts establish—1. The facility with which tubage of the glottis may be performed by fixing a canula on the lower cordæ vocales, which does not interfere with the functions of the epiglottis. 2. The tolerance of this tube by the larynx. 3. The possibility of relieving the asphyxia of croup by this means in preference to tracheotomy. 4. The facility with which large pseudo-membranous concretions formed in the trachea and bronchi may pass through this intra-glottal tube. 5. The utility of this new resource for surgeons residing in remote localities, destitute of all assistance.—*Union Médicale*, No. 110. (*Medical Times and Gazette*, Oct. 30, 1858.)

